# Clinical Features and Voxel-Based-Symptom-Lesion Mapping of Silent Aspiration in Acute Infratentorial Stroke

**DOI:** 10.1007/s00455-023-10611-z

**Published:** 2023-08-03

**Authors:** H. Lesch, M. Wittayer, M. Dias, A. Nick, A. Ebert, P. Eisele, A. Alonso

**Affiliations:** Department of Neurology, Medical Faculty Mannheim, Mannheim Center for Translational Neuroscience (MCTN), University of Heidelberg, Theodor-Kutzer-Ufer 1-3, 68167 Mannheim, Germany

**Keywords:** Stroke, Silent aspiration, Dysphagia, Pneumonia, Infratentorial

## Abstract

Post-stroke dysphagia (PSD) is a severe and common complication after ischemic stroke. The role of silent aspiration as an important contributing factor in the development of a dysphagia-associated complications, in particular aspiration-associated pneumonia has been insufficiently understood. The aim of this study was to investigate the characteristics and risk factors of silent aspiration in patients with acute infratentorial stroke by FEES and to identify culprit lesions in stroke patient with a high risk of silent aspiration via voxel-based-symptom-lesion mapping (VBS/ML). This study is a retrospective observational study based on a prospectively collected FEES and stroke database. Consecutive patient cases with acute ischemic infratentorial stroke and FEES examination between 2017 and 2022 were identified. Group allocation was performed based on PAS scores. Imaging analysis was performed by manual assignment and by VBS/ML. Group comparisons were performed to assess silent aspiration characteristics. Binary logistic regression analysis was performed to determine if baseline clinical, demographic, and imaging parameters were helpful in predicting silent aspiration in patients. In this study 84 patient cases with acute infratentorial stroke who underwent FEES examination were included. Patients were moderately affected at admission (mean NIH-SS score at admission 5.7 SD ± 4.7). Most lesions were found pontine. Overall 40.5% of patients suffered from silent aspiration, most frequently in case of bilateral lesions. Patients with silent aspiration had higher NIH-SS scores at admission (*p* < 0.05), had a more severe swallowing disorder (*p* < 0.05) and were 4.7 times more likely to develop post-stroke pneumonia. Patients who underwent FEES examination later than 72 h after symptom onset were significantly more likely to suffer from silent aspiration and to develop pneumonia compared to patients who underwent FEES examination within the first 72 h (*p* < 0.05). A binary logistic regression model identified NIH-SS at admission as a weak predictor of silent aspiration. Neither in manual assignment of the lesions to brain regions nor in voxel-wise statistic regression any specific region was useful in prediction of silent aspiration. Silent aspiration is common in patients with infratentorial stroke and contributes to the risk for pneumonia. Patients with silent aspiration are more severely affected by stroke, but cannot reliably be identified by NIH-SS at admission or lesion location. Patients suffering from acute infratentorial stroke should been screened and examined for PSD and silent aspiration.

## Introduction

Post-stroke dysphagia (PSD) is a common and formidable complication in patients with acute ischemic stroke. Current studies show an initial dysphagia in at least half of all patients with ischemic or hemorrhagic stroke [1, 2]. More than half of stroke patients with dysphagia aspirate and these patients have an up to fourfold increased risk of developing aspiration pneumonia at an early stage [3–5]. In addition, PSD leads to increased morbidity and mortality due to further complications such as malnutrition and dehydration [6].

While a recent meta-analysis identified a number of relevant risk factors for PSD including aspiration, most studies made insufficient distinctions between silent and non-silent aspiration [7–9]. Silent aspiration, i.e., aspirating saliva or during oral intake of food or liquids endotracheally without triggering sufficient protective reflexes is common after stroke but its implications for PSD as well as dysphagia-associated complications have not been well understood [4, 10, 11].

Putative clinical markers of silent aspiration in the context of swallow screening (SSA) such as a hypophonically weak voice or a hoarse, wet voice or cough are insufficient to reliably detect silent aspiration [12–14]. While some recent findings recommend screening for impaired cough reflex as a tool to detect silent aspiration, no consensus regarding clinical diagnosis of silent aspiration has been established [11]. As a result, silent aspiration often goes undetected and precautions to reduce the risk of aspiration are not taken. The gold standard for detecting silent aspiration is fiberoptic endoscopic evaluation of swallowing (FEES). Despite the widespread availability of the diagnostic FEES and their inclusion in the certification criteria for stroke units in Germany silent aspiration is likely underdiagnosed [15]. This is especially true for infratentorial stroke: While patients may present with only subtle neurological deficits such as mild ataxia, a prevalence of silent aspiration in brainstem infarction of up to 80% has been reported [16, 17].

Even though PSD and silent aspiration after infratentorial stroke is well known and common, the mechanisms leading to silent aspiration are poorly understood and the regions and the types of brain lesions likely to lead to silent aspirations are unknown. To date, there are no comprehensive studies on risk factors for silent aspiration, its frequency and biomarkers for the prediction of silent aspiration in patients with infratentorial stroke.

The aim of this study was to investigate the characteristics of PSD by FEES and to identify risk factors as well as culprit lesions for silent aspiration in patients with acute infratentorial stroke via voxel-based-symptom-lesion mapping (VBS/ML), which is used to establish voxel-wise connections between symptoms and the location of lesioned areas either from continuous variables as multiple correlation relationship or from dichotomous variables with Liebermeister’s test comparing the lesions of two clinically distinct groups. We hypothesized that silent aspiration is common in infratentorial stroke, its occurrence depends on lesion location and is attributed to worse outcome in patients.

## Materials and Methods

### Subjects

Patients were screened by retrospective review of our prospectively collected FEES database. The inclusion criteria were as follows: (i) hospitalization with first-ever ischemic stroke restricted to the mesencephalon, pons, cerebellum, or medulla oblongata verified by magnetic resonance imaging (MRI) (ii) age 18 years or older, and (iii) FEES diagnostics during hospitalization for acute ischemic stroke. The exclusion criteria were as follows: (i) missing medical records or brain MRI scans; (ii) previous history of stroke, dementia, or other diseases that could cause difficulty in swallowing.

**Fig. 1:**
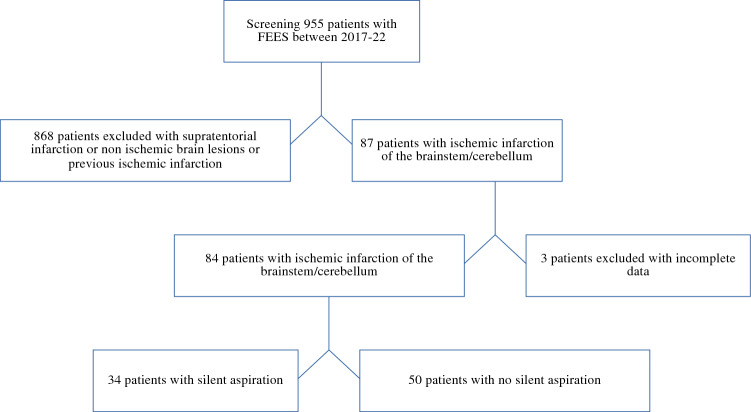
Screening procedure and database. *FEES* fiberoptic endoscopic evaluation of swallowing

Of a total of 955 patients with acute ischemic stroke who were admitted between 2017 and 2022 to the stroke unit of the Medical Faculty Mannheim and underwent FEES diagnostics, 87 had had a first ever ischemic infarction of the brainstem or cerebellum. Ultimately, a total of 84 patients satisfied the criteria and were included for analysis, while 3 had to be excluded due to missing MRI or insufficient clinical data. Data from these patients were collected retrospectively (Fig. 1).

The study was approved by the institutional review board of our institution (2013-813R-MA) and the requirement for informed consent was waived due to the retrospective design of the study.

## Methods

### Review of Medical Records

Pseudonymized patient demographic and clinical characteristics were recorded from the medical records, including age, sex, Glasgow Coma Scale (GCS) [18] at admission, National Institutes of Health Stroke Scale score (NIH-SS) [19] at admission, after 24 h and at discharge, modified Rankin Score (mRS) [20] at admission and at discharge and duration between onset of stroke and FEES (number of days).

Pneumonia was diagnosed using an adapted version of the diagnostic criteria first established by Kropek et al. in accordance with in-house hospital guidelines [21]. Diagnosis of pneumonia required the presence of at least one major criterion (newly formed or progressive pulmonary infiltrate in x-ray or computer tomography consistent with radiographic diagnosis of pneumonia) and at least two minor criteria (Hyperthermia > 38.0 °C, newly developed purulent sputum, new respiratory distress, identification of pathogen in bronchoscopy or pleural puncture, concordant pathogen in bacteremia, polymerase chain reaction (PCR) detection of virus or antigen in respiratory secretions).

### Fiberoptic Evaluation of Swallowing Study

Physicians or dysphagia specialists of the Department of Neurology conducted the FEES according to a modified version of Langmores’ procedure following an in-house procedural manual in accordance with the recommendations of the German Society of Neurology (DGN) [22]. Briefly, subjects were seated, after insertion of the fiberoptic laryngoscope the secretion status was evaluated, and the patient was successively given standard volumes of liquids and food with increasing degree of solidity. Penetration and aspiration was assessed via the Penetration-aspiration scale (PAS) scoring penetration of a saliva, liquids or solids into the airway and elucidated protective measure from 1 to 8 [23]. A PAS of 1 describes no penetration of material into the airway, scores between 2 and 7 are given in case of penetration into the airway triggering protective measures to clear said material from the airway and a score of 8 is given in case of observed aspiration of material below the vocal cords without any effort by the patient, such as coughing, to eject material. Highest PAS score, representing worst reaction to stimuli is then recorded. The severity of dysphagia was assessed by Bogenhausener Dysphagie Score (BODS) [24]. BOD Score consists of two eight-level scores, with BODS-1 ranging from 1 to 8 indicating difficulty in swallowing saliva during FEES examination, 1 meaning no difficulty swallowing saliva, whereas 8 is attributed to severe difficulty in swallowing saliva requiring saliva management via tracheostomy tube. BODS-2 grades impairment in food intake from 1 to 8 during FEES examination, 1 reflecting no difficulty swallowing food in FEES examination, whereas 8 indicates severe impairment requiring nutrition via nasogastric tube or intravenously. The sum of BODS-1 and BODS-2 builds the BOD Score total reflecting the degree of severity of dysphagia. Secretion status was graded via Murray Secretion Scale (MSS) [25]. Patients with MSS of 0 have no observable saliva accumulation; grade 1 depicts saliva pooling in the vallecula and the pyriform sinus. Presence of laryngeal saliva pools represents grade 2 and patients with saliva in the laryngeal vestibule, that remains after spontaneous swallowing, are given grade 3.

### Imaging Analysis

Lesion location was determined by a single rater experienced in neuroradiology (HL) and subsequently independently confirmed by a second rater experienced in neuroradiology (MW). Brain lesion location was defined as mesencephalic, pontine, cerebellar, medullary, and multilocular. Infarction side was defined as left, right, or bilateral. White matter lesions in MRI were scored according to Fazekas scale [26]. Fazekas scale assigns a grade from zero to three for white matter lesion in periventricular and deep white matter according to the size and confluence of lesions, representing the absence (0) of or mild (1), moderate (2) or severe (3) degree of white matter lesions.

To determine lesion volume, binary lesion masks were produced manually by segmenting the lesion area on all consecutive sections that displayed the lesion. All lesions were mapped using ITK-SNAP software (http://www.mccauslandcenter.sc.edu/mricro/mricron/) and were drawn manually on individual diffusion-weighted imaging (DWI) scans by a single researcher (HL) and verified in a second step by a second researcher (MW). Fluid-attenuated inversion recovery (FLAIR) hyperintensities with no corresponding diffusion-restriction, representing leukoaraiosis or silent chronic stroke lesions (with no corresponding DWI lesion), were not included in stroke lesion segmentation. DWI images were co-registered with the same subject’s T1 sequences and the resulting transformation matrices were applied to the binary lesion maps using SPM12 coregistration algorithm (Wellcome Department of Neuroscience, London, UK; http://www.fil.ion.ucl.ac.uk/spm/software/spm12/). The T1-maps were warped to MNI-space using the normalizing algorithm of SPM 12. The resulting transformation matrices were applied to the coregistered binary lesion maps. Normalized lesions were fed into voxel-wise statistical mapping analysis using VBS/ML algorithms as implemented in the SVRLS- Toolbox ([27]; https://github.com/atdemarco/svrlsmgui). Regression data was corrected for lesion volume. Support-vector-regression performed and thresholds were adjusted using 1000 permutations.

Lesions of dichotomous clinical groups were examined using Liebermeister’s Test implemented in the NiiStat- Toolbox (McCausland Center for Brain Imaging, University of South Carolina, https://github.com/neurolabusc/NiiStat). Both toolboxes were run on Matlab (R2020b, The MathWorks Inc., Natick, MA, USA) and SPM 12.

### Statistical Analysis

Descriptive statistics were used to present the clinical data. The patients were divided into two groups based on FEES results. Group allocation was performed using PAS score, with a PAS score of 8 indicating silent aspiration. Patients with PAS = 8 were attributed to the ‘silent aspiration group,’ whereas patients with PAS ≤ 7 were classified into the ‘no silent aspiration group.’

*T* test were performed for parametric variables (e.g., age) while Mann–Whitney U test was conducted for non-parametric variables (e.g., NIH-SS, mRS, GCS, Fazekas) for comparative analysis of the two groups. *Χ*^2^-test or Fisher’s Exact Test (FET) was used to compare categorical variables as appropriate. We performed a binary logistic regression analysis to determine if baseline clinical (NIH-SS at admission), demographic (sex and age) and imaging parameters (lesion location (mesencephalic, cerebellar, pontine, medullar, or multilocular) and chronic ischemic changes (Fazekas- grading (using dichotomized Fazekas scores, with Fazekas score 0 and 1 grouped as minor white matter lesions and Fazekas score 2 and 3 grouped as relevant white matter lesions)) were significant factors in the prediction of silent aspiration. We used Enter as the method to perform binary logistic regression. Exponent(B) and its 95% confidence intervals as well as equivalent *p* values are reported. The SPSS statistical package (IBM SPSS Statistics for Windows, Version 21.0; IBM, Armonk, NY, USA) was used for all statistical analyses. Statistical significance was defined as *p* < 0.05.

## Results

### Baseline Characteristics

General demographic characteristics and clinical variables of the patients are listed in Tables 1 and 2. Of the 84 patients, 62 (73.8%) were male, the age ranged from 28 to 94 years (mean = 70.0, SD ± 14.0). None of the patients had an endotracheal tube or had previously been tracheotomized with placement of a tracheal tube to prevent aspiration. FEES was performed at a mean of 3.8 days (SD ± 3.8) after admission with a minimum of 0 and a maximum of 20 days. Silent aspiration was detected in 34 (40.5%) patients during the FEES examination. Overall 24 (28.6%) patients were diagnosed with post-stroke pneumonia during their treatment for acute ischemic stroke.

**Table 1:** Patient characteristics

Characteristics	Total cohort	Patients with no silent aspiration	Patients with silent aspiration	*p* value
General features				
Number of patients, *n*	84	50 (59.5%)	34 (40.5%)	–
Age, mean ± SD	70.0 ± 14.0	72.7 ± 11.6	68.1 ± 15.2	0.89
Gender, male, *n* (%)	62 (73.8%)	39 (78%)	23 (67%)	0.21
Silent aspiration, *n* (%)	34 (40.5%)	59.5%	40.5%	–
Nasogastric tube (%)	18 (21.4%)	8 (16%)	10 (29.4%)	0.18
Fazekas, median (IQR)	1 (0; 2)	1 (0; 2)	1 (1; 2)	0.74
Onset until FEES (days) mean ± SD	3.8 ± 3.8	3.2 ± 3.7	4.7 ± 3.8	0.08
Clinical features				
NIH-SS admission, mean ± SD	5.7 ± 4.7	4.6 ± 2.8	7.4 ± 6.3	< 0.05
NIH-SS after 24 h, mean ± SD	5.8 ± 3.6	4.7 ± 3.1	7.6 ± 3.6	< 0.05
NIH-SS discharge, mean ± SD	4.7 ± 3.5	4.2 ± 3.5	5.4 ± 3.4	0.06
mRS admission, mean ± SD	3.6 ± 1.3	3.2 ± 1.4	4.2 ± 1.0	< 0.05
mRS discharge, mean ± SD	3.2 ± 1.4	3.0 ± 1.5	3.6 ± 1.2	0.12
GCS, mean ± SD	14.8 ± 1.2	15.0 ± 0.2	14.5 ± 1.6	0.13
Pneumonia, *n* (%)	24 (28.6%)	8 (16%)	16 (47.1%)	< 0.05
Dysphagia				
MSS, median (IQR)	1 (0; 2)	1 (0; 1)	1 (1; 3)	< 0.05
BODS total, median (IQR)	6 (5; 9)	5 (3; 8)	9 (5; 10)	< 0.05
BODS 1, median (IQR)	1 (1; 2)	1 (1; 2)	2 (1; 3)	< 0.05
BODS 2, median (IQR)	4 (3; 7)	4 (2; 7)	7 (4; 8)	< 0.05
PAS, median (IQR)	7 (3; 8)	3 (2; 5)	8 (8; 8)	–

*BODS* Bogenhausener Dysphagie Score, *FEES* fiberoptic endoscopic evaluation of swallowing, *GCS* Glasgow Coma Scale, *IQR* interquartile range, *mRS* modified Rankin score, *MMS* Murray Secretion Scale, *NIH-SS* National Institutes of Health Stroke Scale, *PAS* penetration-aspiration-Scale, *SD* standard deviation

**Table 2:** Lesion location and lesion volume

Lesion location	All patients (*n* = 84)	Patients with no silent aspiration (*n* = 50)	Patients with silent aspiration (*n* = 34)	*p* value
Pontine	46 (54%)	25 (50%)	21 (61.8%)	0.40
Cerebellar	17 (20.2%)	11 (22%)	6 (17.6%)	0.83
Mesencephalic	2 (2.4%)	1 (2%)	1 (2.9%)	0.65
Medullar	7 (8.3%)	6 (12%)	1 (2.9%)	0.28
Multiple locations	12 (14.3%)	7 (14%)	5 (14.7%)	0.82
Infarction side				
Right	40 (47.6%)	23 (46%)	17 (50%)	0.89
Left	34 (40.5%)	22 (44%)	12 (35.3%)	0.56
Bilateral	10 (11.9%)	5 (10%)	5 (14.7%)	0.76
Lesion volume median (IQR) (mm^3^)	–	821 (442; 1854)	1335 (679; 3767)	0.13

The table shows number of patients with lesion in specific region, brackets represent percentage of lesion location within silent aspiration group or no silent aspiration group. P-values represent comparison of specific lesion location of silent aspiration group vs. no silent aspiration group

*IQR* interquartile range, *mm*^*3*^ millimetres cubed

### Group Comparison Silent Aspiration vs. No Silent Aspiration

Differences between patients with silent aspiration (PAS = 8) and patients without (PAS ≤ 7) are shown in Table 1. Patients with silent aspiration had a significantly higher symptom burden at admission than patients without silent aspiration as reflected by significantly higher NIH-SS scores at admission (*U* = 577.500; *Z* = − 2.504, *p* < 0.05) as well as higher mRS at admission (*U* = 531.000; *Z* = − 3.004, *p* < 0.05). Severity of dysphagia scored by BODS was significantly higher in patients with silent aspiration (*U* = 446,000; *Z* = − 3.719, *p* < 0.05). Patients with silent aspiration had significantly higher initial secretion status score upon FEES examination graded via MSS (*U* = 610.500; *Z* = − 2.295, *p* < 0.05).

Out of the 34 patients with silent aspiration 16 developed aspiration pneumonia, while in the no silent aspiration group out of 50 patients 8 developed post-stroke pneumonia (*χ*^2^ (1, *N* = 84) = 9.566, *p* < 0.05). Patients with silent aspiration were 4.67 more likely to develop post-stroke pneumonia (OR 4.7, 95% CI [1.7, 12.6]).

BODS scores for patients with or without pneumonia are shown in Table 3. In the no silent aspiration group, BODS total, BODS-1 and BODS-2 were significantly higher for patients who developed pneumonia (*U* = 63.500; *Z* = − 2.794, *p* < 0.05); (*U* = 92.000; *Z* = − 2.621, *p* < 0.05); (*U* = 77.000; *Z* = − 2.439, *p* < 0.05). In the silent aspiration group BODS showed a trend but ultimately did not differ significantly between patients, who developed pneumonia and patients who did not (*U* = 89.000; *Z* = − 1.929, *p* = 0.06); (*U* = 109.500; *Z* = − 1.276, *p* = 0.24); (*U* = 88.500; *Z* = − 2.000, *p* = 0.06).

**Table 3:** Dysphagia scores in patients with pneumonia

Pneumonia	Total cohort (*n* = 84)			No silent aspiration (*n* = 50)			Silent aspiration (*n* = 34)		
	Yes (*n* = 24)	No (*n* = 60)	*p* value	Yes (*n* = 8)	No (*n* = 42)	*p* value	Yes (*n* = 16)	No (*n* = 18)	*p* value
BODS 1, median (IQR)	2 (1;3)	1 (1; 2)	< 0.05	2 (1; 3)	1 (1;1)	< 0.05	2 (1; 3)	2 (1; 2)	0.24
BODS 2, median (IQR)	7 (4; 8)	4 (3; 7)	< 0.05	7 (4, 8)	3 (2; 6)	< 0.05	7 (5; 8)	4 (4; 7)	0.06
BODS total, median (IQR)	9 (7; 10)	5 (4; 8)	< 0.05	8 (6; 10)	5 (3; 7)	< 0.05	10 (7; 10)	6 (5; 9)	0.06

*IQR* interquartile range, *BODS* Bogenhausener Dysphagie Score

There were no significant differences in the two groups for the following variables at baseline: age, sex, GCS at admission and white matter lesions scored according to Fazekas.

Nutrition via nasogastric tube was also neither associated with detection of silent aspiration nor with the occurrence of pneumonia. Both within the silent aspiration group and within the non-silent aspiration group, NIH-SS scores at admission and mRS scores at admission did not differ significantly between patients who developed pneumonia and those who did not.

We compared patients who underwent early FEES (defined as within 72 h of admission) with patients on whom FEES was performed > 72 h after admission. Patients with late FEES were significantly more likely to suffer from silent aspiration (*χ*^2^ (1, *N* = 84) = 6.686, *p* < 0.05) and to develop pneumonia (*χ*^2^ (1, *N* = 84) = 4.887, *p* < 0.05) with a 3.3 fold increased risk for silent aspiration (OR 3.3, 95% CI [1.3, 8.1]) and a threefold increased risk for pneumonia (OR 3.0, 95% CI [1.1, 8.1]).

### Predictors of Silent Aspiration

In order to determine if baseline clinical (NIH-SS at admission), demographic (sex and age) and imaging parameters (lesion location and chronic ischemic changes) were significant factors in predicting silent aspiration in patients, we performed a binary logistic regression analysis.

The model revealed NIH-SS at admission as a significant predictor for silent aspiration (*p* < 0.05, Exp (B) = 1.2, CI [1.0, 1.4]).

### Lesion Location Association with Silent Aspiration

**Fig. 2:**
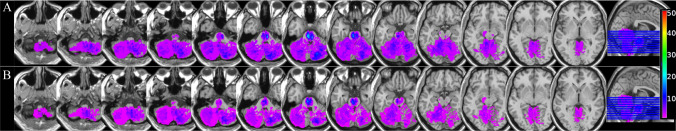
Heat maps representing lesion location in axial MRI for patients with silent aspiration (**A**) and patients without silent aspiration (**B**). Intensity of color reflects the number of overlapping patients. *MRI* magnetic resonance imaging

The distribution of infarct locations by manual assignment of lesions to brain regions is shown in Table 2. The most frequent infarct location was pontine (Fig. 2). While 45.7% of patients with a pontine lesion had silent aspiration diagnosed during FEES examination, there was no significant difference between infarct location in patients with silent aspiration compared to patients with no silent aspiration (*χ*^2^ (4, *N* = 84) = 2.776, *p* = 0.61; FET). Infarct side also did not yield a significant difference between the two groups, but out of the 10 patients with bilateral infarction, 5 suffered from silent aspiration, leading to the highest relative number of patients with silent aspiration in this group (*χ*^2^ (2, *N* = 84) = 1.295, *p* = 0.54; FET).

There was no significant difference of the normalized lesion volumes between patients with and without silent aspiration (Median patients with silent aspiration 1334.5 mm^3^; IQR 679 mm^3^; 3767 mm^3^, median patients without silent aspiration: 821 mm^3^; IQR 442 mm^3^; 1854 mm^3^; (*U* = 63.500; *Z* = − 2.794, *p* = 0.16) as shown in Table 2. In the voxel-wise imaging analysis using Liebermeister’s Test to compare the lesions of clinical groups with and without silent aspiration did not reveal any significant voxel-wise differences between the specific lesion localization and the presence of silent aspiration or the development of pneumonia. Regressional VBS/ML analysis did not reveal any significant voxel-wise lesion—score interaction for BODS and its subscales as well as PAS and Murray scores using clusterwise permutation correction.

## Discussion

In our study we compared 84 patients suffering from acute infratentorial stroke regarding clinical characteristics and complications with a focus on silent aspiration as a potential detriment in recovery following stroke.

Post-stroke complications in general and PSD in particular are known risk factors for increased morbidity and mortality following ischemic stroke and dysphagia management is essential in the treatment of patients suffering from acute stroke.

This study further underlines the risks of undiagnosed PSD and need for dysphagia diagnostics via FEES examination in patients with infratentorial stroke. Our findings show a high incidence (40.5%) of silent aspiration in patients suffering from acute infratentorial stroke exceeding previously reported rates (2–25%) [17]. In our study these patients presented a significantly higher risk of developing pneumonia by aspiration than patients without silent aspiration (47.1% vs. 16%).

### Lesion Location as a Predictor for Dysphagia and Silent Aspiration?

Previous studies have established that either infra- and supratentorial brain lesions contribute to PSD, but there is some evidence that brain stem lesions are more likely to cause clinically relevant dysphagia compared to strokes of the anterior circulation [28]. Lesions in the brainstem are more likely to involve deficits in motor control of swallowing while supratentorial lesions may affect sensory perception and the cerebellum contributes to coordination and regulation of the swallowing motion [29–33]. In our data infarct volumes did not show a significant difference between patients with silent aspiration and patients without. We estimated lesion location to be more important than lesion size in infratentorial stroke, but neither in manual assignment of the lesions to brain regions nor in voxel-wise statistic regression any specific region could be detected rendering patients more susceptible to silent aspiration. Especially no significant voxel-wise differences were found between the silent aspiration group compared to the no silent aspiration group. Moreover, there were no significant voxels that showed a correlational interaction with clinical dysphagia scores.

The failure to discern a particular region that is more often lesioned in patients with silent aspiration can be explained by the complex pathophysiology of silent aspiration, involving multitude of different areas of the brain corresponding to sensory and motor control, cranial nerve nuclei involvement and the relevant interaction between different regions. Consequently, it has been shown, that multifocal lesions result in the highest risk for developing PSD [29]. In our collective the highest percentage of silent aspiration was detected in patients with bilateral lesions. Our results therefore do not allow for selecting patients by their lesion location for further diagnostics for dysphagia and rather stipulate caution in all patients with brainstem stroke and warrant the need for FEES examination.

### NIH-SS as a Predictor for Dysphagia

Previous studies have proposed using NIH-SS and by that, severity of disability at admission as a tool to predict severity of dysphagia and risk for complications as stroke severity has been shown to increase risk of PSD and risk for aspiration pneumonia [34–38]. In our study patients with silent aspiration were on average more severely affected by their respective stroke than patients without silent aspiration compatible with these previous results. Congruously, in our prediction model for silent aspiration NIH-SS at admission contributed to the prediction of silent aspiration but the detected relationship was not strong and seems insufficient as a sole tool to predict silent aspiration. This limitation of NIH-SS becomes even more evident, since beforementioned publications strongly varied in recommended cut-offs in the NIH-SS triggering need for dysphagia screening between low cut offs starting at 5 points and going up to 12 points on the NIH-SS [34–39].

We suspect using NIH-SS as an overall predictor for PSD likely omits mildly affected patients with low NIH-SS scores but with a high risk for pneumonia due to silent aspiration. It seems especially unprecise for patients with infratentorial stroke, as NIH-SS underestimates stroke severity by infratentorial stroke [40]. A retrospective study by Labeit et al. tried predicting via NIH-SS specifically for posterior circulation stroke, suggesting a NIH-SS score of ≥ 5 as a cut off for dysphagia screening in infratentorial stroke with albeit limitations regarding sensitivity and specificity rendering it unsuitable as a sole screening parameter [36]. In our population mean (and median) NIH-SS scores in the silent aspiration were above the aforementioned cut-off of 5 while NIH-SS scores in patients without silent aspiration were below 5. Nonetheless PSD does not automatically result in aspiration pneumonia and patients suffering from silent aspiration might be at higher risk for pneumonia not reflected in the NIH-SS since NIH-SS did not differ in the silent aspiration group between patients with pneumonia and patients who did not suffer from pneumonia. A reason for this discrepancy could lie in the design of the NIH-SS which attributes high scores for weakened motor function of the extremities and speech disability while impairment of the nuclei of the cranial nerves involved in swallowing are underrepresented. Another contributing factor may be that bedridden patients are at higher risk for infections in general and not all hospital acquired pneumonias are the result of aspiration or difficulty swallowing even in stroke patients.

### BODS as a Predictor for Pneumonia

While national guidelines recommend early screening of stroke patients before oral intake (administration of fluids, food, or tablets), patients with mild strokes often only get screened clinically or no documented screening for dysphagia at all [3, 41]. Dysphagia screening and scoring of dysphagia severity via BODS provided a tool in assessing the risk of pneumonia as BODS were higher when looking at all patients with pneumonia compared to patients, who did not develop pneumonia. While BODS total as well as BODS-1 and BODS-2 individually were significantly higher for patient in the silent aspiration group, BODS interestingly was not helpful to determine the risk for pneumonia in the silent aspiration group as scores did not differ in between patients with pneumonia and patients who didn’t suffer this complication in the silent aspiration group.

Therefore screening and early PSD diagnosis within 24 h of admission should be performed routinely and might help to lower the risk of stroke-related pneumonia [42, 43].

Interestingly, the detection rate of silent aspiration was higher in late examination of FEES in our cohort. This could be representative of late brain swelling and edema often observed in infratentorial ischemic stroke, especially cerebellar stroke, occurring most commonly after two days up to six days, as previously reported, and could indicate the necessity of reevaluation of dysphagia in patients at risk [44–46].

### Limitations

The interpretation of our results is limited by the retrospective study design of acquiring data from an internal databank with a limited number of patients. Patients in this study were only included if they had received FEES examination, which may have resulted in selection bias where patients, who showed clinical signs of dysphagia or were estimated to be at risk of pneumonia were more likely to undergo dysphagia diagnostics. Patients with prior stroke or prior documented dysphagia were excluded. In order to identify risk factors for silent aspiration and biomarkers allowing the prediction of silent aspiration further prospective studies are needed.

## Conclusion

The results of this study suggest that patients with acute infratentorial stroke are at high risk for silent aspiration, irrespective of the lesion location and NIH-SS scores. Patients suffering from silent aspiration, in turn, are an at-risk population for severe post-stroke complications, especially pneumonia, deteriorating their outcome and increasing morbidity and mortality in affected patients. Clinicians and health administrators should be aware of the critical need to identify patients with potential silent aspiration regardless of stroke severity and implement early as well as specialized dysphagia diagnostics.

## Data Availability

The datasets generated during and analyzed during the current study are available from the corresponding author on reasonable request.
